# Elucidating Gene Functions in Congenital Heart Disease

**DOI:** 10.1007/s11936-026-01157-y

**Published:** 2026-10-06

**Authors:** Sukanya Raghu, Ankit K. Tamta, Sanchita Sanchaya Dey, Mohamed Imdhiyas Abdul Basheed, Aniqua Tasnim Chowdhury, Swetansu K. Hota, Nathan J. VanDusen

**Affiliations:** Department of Pediatrics, Herman B Wells Center for Pediatric Research, Indiana University School of Medicine, Indianapolis, IN 46202 USA

**Keywords:** Congenital Heart Defects, Cardiac Development, Transcription, Functional Genomics, Genome Editing.

## Abstract

**Purpose of Review:**

Congenital heart defects (CHDs) arise from disruption of precisely orchestrated developmental programs that coordinate cardiac lineage specification, morphogenesis, maturation, and tissue remodeling. This review highlights recent advances in experimental systems used to connect CHD-associated genes and variants to developmental mechanisms.

**Recent Findings:**

In vivo mouse and vertebrate studies have refined models of early cardiogenic mesoderm formation, heart-field allocation, valve development, outflow tract morphogenesis, epicardial-myocardial interactions, and ventricular compaction. Parallel work on postnatal cardiac maturation highlights how RNA processing, metabolic remodeling, immune signaling, and non-myocyte populations influence cardiomyocyte maturation and regenerative competence, with implications for long-term outcomes in CHD survivors. Human pluripotent stem cell models provide complementary platforms for investigating gene functions: two-dimensional differentiation enables scalable, temporally controlled analysis of lineage commitment and cell-autonomous phenotypes, whereas organoids and other three-dimensional models introduce spatial organization, multicellular interactions, and tissue-level readouts. Genome editing, CRISPR screening, base and prime editing, regulatory-element assays, and inducible protein-depletion systems now allow increasingly precise and high-throughput characterization of coding and noncoding variants.

**Summary:**

Together, these advances are enabling a transition in CHD research from descriptive genomics to integrated, multi-model approaches that connect genetic variation to gene function, developmental mechanisms, and disease phenotypes.

## Opinion Statement

Congenital heart defects (CHDs) remain the leading cause of infant mortality attributable to birth defects. However, advances in surgical and medical care have markedly improved survival, creating a large and growing population of CHD survivors who face substantial lifelong ongoing medical needs, and poor long-term cardiovascular outcomes. Increased incorporation of DNA sequencing technologies into clinical workflows is rapidly expanding the number of candidate CHD genes and variants, but translating these genetic discoveries into biological mechanisms remains a major challenge. Cardiac development depends on precisely timed and spatially coordinated events, making embryonic cardiogenesis particularly delicate and difficult to interrogate. Historically, mechanistic studies have also been constrained by the cost and complexity of generating genetically modified animal models, limited access to developing human cardiac tissues, and the absence of scalable human experimental systems.

There are now strong reasons for optimism. Larger genetic studies are improving candidate-gene discovery, while CRISPR-based genome editing has greatly simplified the generation of engineered models. Human iPSC-based cardiac differentiation systems have become widely accessible, enabling direct assessment of human gene function and systematic comparison with findings from non-human models. Cardiac organoids increasingly capture aspects of three-dimensional tissue organization, while scalable in vitro systems enable CRISPR perturbation screens, massively parallel regulatory-element assays, and systematic investigation of environmental, chemical, hormonal, and biomechanical influences on cardiogenesis. Furthermore, newly developed AI-powered tools are already accelerating experimental design, data generation, and data analysis. In coming years, AI models integrating high-dimensional datasets across assays, species, and model systems may reveal developmental relationships, prioritize mechanisms, and assist in generating new hypotheses. High-impact future studies will likely pair AI-assisted large-scale data analysis with rigorous genome-editing-based functional experiments across complementary model systems. Collectively, these advances will connect CHD-associated genes to their functions and mechanisms, deepening our understanding of CHD and enabling new therapeutic strategies.

## Introduction

CHDs are a leading cause of infant mortality and the leading cause of infant mortality due to birth defects [1]. Proper cardiac morphogenesis requires the coordinated specification, migration, differentiation, and remodeling of multiple progenitor and differentiated cell populations. Since these processes unfold rapidly and depend on precise spatial and temporal regulation, CHD-associated variants can act through diverse mechanisms, including altered lineage allocation, impaired tissue morphogenesis, abnormal signaling, disrupted chromatin or RNA regulation, and defective postnatal maturation.

Recent advances have created an increasingly powerful collection of tools for connecting CHD genetics to developmental mechanisms. In vivo mouse, zebrafish, and other vertebrate models remain essential for defining how candidate genes affect embryonic patterning, hemodynamics, chamber formation, valve development, outflow tract remodeling, epicardial function, and ventricular compaction. However, human pluripotent stem cell systems now allow investigators to conduct parallel studies of patient-specific or engineered alleles in controlled developmental contexts. Two-dimensional differentiation models provide reliable platforms for testing lineage commitment and cell-autonomous phenotypes, whereas organoids and other three-dimensional model systems add spatial organization, multicellular interactions, and tissue-level readouts that better approximate some features of early human cardiogenesis.

Genome editing and functional genomics have further transformed the field by enabling precise allele engineering, coding and noncoding variant testing, CRISPR screens, single-cell perturbation assays, regulatory-element analysis, and timed protein depletion. These approaches make it possible to move beyond gene discovery toward mechanistic interpretation. Here, we review recent progress across in vivo models, postnatal maturation studies, two- and three-dimensional stem-cell platforms, and genome-editing technologies, emphasizing how integrated cross-model strategies can link genotype, cell state, developmental timing, and tissue architecture in CHD.

## In vivo mouse and vertebrate models of early heart development

The heart develops from primitive-streak mesodermal progenitors that migrate to the anterior lateral plate mesoderm to form the cardiac crescent [2, 3]. Lineage tracing shows that myocardial and endocardial progenitors arise from independent primitive-streak lineages before forming the linear heart tube, whereas the epicardium develops later from the proepicardium [4]. The cardiac crescent contains the first heart field (FHF), which primarily forms the left ventricle, part of the atrioventricular canal, and left atrium, and the second heart field (SHF), which contributes to the right ventricle, outflow tract, inflow tract, and most of the atria. The adjacent juxtacardiac field (JCF) contributes to cardiomyocytes and the proepicardium, which produces epicardium, cardiac fibroblasts, and coronary vascular smooth muscle cells [5].

Multiple progenitors and regulatory programs shape cardiogenic mesoderm. An epiblast-derived CX3CR1 + progenitor population emerging before gastrulation contributes to cardiomyocytes and endothelial cells [6]. HAND2 promotes early lineage progression by regulating PDGFRA-dependent cardiac precursor migration and morphogenesis [7]. Retinoic acid-YAP1 signaling activates atrial enhancers and promotes SHF atrial fate while suppressing ventricular identity [8]. EZH2 maintains Isl1 + multipotent progenitor competence and supports SHF differentiation [9]. Thus, cardiogenic lineage allocation integrates progenitor specification, transcriptional and epigenetic programs, and signaling.

Endocardial and valve development requires lineage specification, biomechanical signaling, and myocardial communication. Hemodynamic forces regulate valve morphogenesis, with endocardial primary cilia sensing flow and promoting EndoMT during cushion formation [10]. Myocardial contraction shapes endocardial gene expression, including zebrafish id2b induction, which coordinates myocardial contractility and valve development [11]. Bicuspid aortic valve genomics reveal a substantial polygenic contribution, indicating that disruption of endocardial specification, mechanotransduction, and EndoMT pathways can converge in congenital valve disease [12].

Cardiopharyngeal mesoderm (CPM) and SHF progenitors drive heart-tube elongation and outflow tract (OFT) formation. TBX1 stabilizes CPM identity and coordinates pharyngeal morphogenesis through focal adhesion dynamics and paxillin-dependent migration [13]. TBX1 also interacts with retinoic acid-responsive pathways, including six family genes, to control cardiomyocyte formation and SHF deployment [14]. In the SHF, SOX9 is required for OFT septation and semilunar valve formation [15]. Arterial valve progenitors arise directly from SHF cells, linking SHF specification to valve morphogenesis [16]. Together, restricted progenitors coordinate chamber specification and OFT morphogenesis.

After chamber specification, myocardium-epicardium interactions drive ventricular maturation, coronary formation, and compaction. The epicardium supplies epicardial-derived cells (EPDCs) and a signaling niche for myocardial growth and vascular development. Epicardial-derived fibroblasts regulate myocardial expansion and coronary vascular remodeling through collagen-dependent signaling [17], and in vitro EPDC models define differentiation trajectories and signaling properties [18]. EPDC invasion into trabecular myocardium is controlled by NFP-FGF signaling required for ventricular compaction and compact wall formation [19]. MIR503HG disruption perturbs NOTCH signaling, causing left ventricular non-compaction cardiomyopathy and showing how lncRNAs preserve ventricular architecture [20].

Later morphogenesis requires temporal coordination of cardiomyocyte proliferation, differentiation, metabolism, and remodeling through dosage-sensitive regulators. Ventricular chamber morphogenesis depends on a narrow window of PKN2 activity [21]. TBX5 dosage is required for interventricular septum development, and its loss causes ventricular septal defects [22]. ASNA1 maintains tail-anchored protein stability and vesicular trafficking, whereas B-RAF supports myocardial growth and function [23, 24]. Post-transcriptional regulation is also critical: DDX3X directs translation of structured cardiac mRNAs in a female-specific program, and RBPMS/RBPMS2 safeguard alternative splicing for cardiomyocyte maturation [25, 26]. These mechanisms converge with evidence that mitochondrial metabolism, RNA processing, chromatin regulation, and cytoskeletal organization function as integrated checkpoints [27–30]. Thus, transcription factors establish lineage identity, epigenetic and RNA mechanisms stabilize programs, and metabolic/protein networks execute morphogenesis.

Single-cell, spatial transcriptomic, and high-content imaging platforms now assist in mapping candidate-gene functions to embryonic heart structures. These atlases place cardiac progenitors, myocardial subtypes, epicardial derivatives, and vascular populations in dynamic signaling niches, helping interpret mutant morphogenesis. Systems genetics has identified proteasome factors and other candidate regulators, while integrated single-cell and spatial analyses have clarified early progenitor origins and deployment cues [31–33]. Similar approaches link genes to OFT development [34], ventricular compaction [35], coronary growth [36], and conduction system patterning [37], connecting perturbations to affected structures. Developmental cardiology is therefore moving from lineage description toward genotype–cell-state–architecture maps.

Together, these studies bridge human genetics to mechanisms in model systems. CHD, cardiomyopathy, and syndromic variants can be interpreted against atlases that map genes to cardiogenic mesoderm, SHF, epicardium, myocardium, or valve progenitors. Studies of GATA4, NCKAP1, SOX4, and trisomy 21 illustrate how human genetic findings translate into altered progenitor behavior, myocardial reprogramming, disrupted signaling, and morphogenesis [38–41]. Broader CHD sequencing continues to uncover recessive and de novo contributors for functional testing. These efforts create a bidirectional pipeline in which atlases inform variant interpretation, and human genetics identifies pathways that can be traced to embryonic cell populations and morphogenetic events.

## Postnatal Maturation and Regenerative Competence

Surgical correction of structural heart defects has created a growing population of pediatric and adult CHD survivors. Although these defects may be anatomically repaired, patients retain CHD-associated mutations that may influence postnatal processes such as cardiomyocyte maturation and, ultimately, long-term cardiac outcomes. Postnatal heart maturation is a coordinated transition across cardiac cell types, with RNA processing emerging as a central regulatory mechanism. Recent work shows that RBP-mediated alternative splicing broadly regulates postnatal heart maturation in mice; overexpression of RBPs such as NCBP2 represses this process, emphasizing the need for precise control of the splicing landscape [42]. This is highly relevant to CHD genes, many of which encode dosage-sensitive transcription factors, chromatin regulators, or RNA-binding proteins whose functions may depend on developmental timing and splice isoform usage.

Single-cell and spatial transcriptomic studies further show that cardiomyocyte maturation is heterogeneous across the developing heart and does not proceed through discrete stages [43]. Transitional cardiomyocyte states may therefore represent therapeutic windows for regenerative or maturation-directed interventions. Complementary single-cell functional genomics approaches are beginning to define the regulatory networks controlling these transitions. For example, Wang et al. (2025) integrated single-nucleus and spatial transcriptomics to generate a temporal and spatial atlas of postnatal heart development and used PIP-seq to identify 21 candidate regulators of cardiomyocyte maturation [44]. Similarly, studies of Negative elongation factor A (NelfA) highlight the importance of transcriptional and RNA-processing control, as cardiac-specific NelfA deficiency caused cardiomyopathy by 2–3 months of age [45].

Metabolic remodeling is another essential feature of postnatal cardiomyocyte maturation, marked by a shift from glycolysis toward mitochondrial oxidative metabolism. PKM1 supports embryonic cardiomyocyte proliferation by regulating energy status and NFYα stability [46], while MitoSAM-dependent lipoylation promotes postnatal metabolic remodeling and TCA-cycle function [47]. Mitochondrial positioning is also critical: loss of RHOT1 or RHOT2 disrupts mitochondrial organization, sarcomere structure, and cardiac function [48]. Together, these studies support a model in which genetic, transcriptional, RNA-processing, and metabolic programs converge during postnatal cardiomyocyte maturation to establish durable cardiac performance.

The mature mammalian heart has limited regenerative capacity, whereas neonatal cardiomyocytes (CMs) can proliferate and regenerate after injury. This ability declines during postnatal development as cell-cycle regulation, dedifferentiation, immune signaling, and epigenomic constraints converge to restrict CM proliferation. Recent studies identify immune–epigenetic regulation as one mechanism: CD4 + regulatory T cells promote expression of MRG15, which complexes with TIP60, p300, and RNA polymerase II to activate genes such as Ccnd1 and drive G1-to-S progression [49]. However, postnatal alternative splicing of MRG15 promotes accumulation of the long isoform, MRG15L, contributing to senescence and reduced regenerative potential [50].

RNA processing and transcriptional regulation also shape regenerative competence. SRSF3-mediated splicing regulates epicardial proliferation [51], LAP2a enhances CM proliferative capacity [52], and RBM22 promotes regeneration by increasing transcriptional accessibility [53]. Hnrnpa1 supports daughter CM formation and repair after myocardial infarction by regulating dedifferentiation and cell-cycle dynamics through Mettl3 splicing [54]. In contrast, Foxp1 suppresses proliferation after adult MI and neonatal apical resection through the USP20-HIF1α-Hand1 pathway [55], while SMARCE1 also restrains CM proliferation [56].

Cardiac maturation and regenerative decline are further influenced by non-myocyte populations and structural remodeling. Ribosomal protein defects impair CM proliferation and may contribute to hypoplastic left heart syndrome [57]. Cardiac fibroblast activity, activation, and self-renewal depend on the lncRNA Charme [58], while PKR1–miR-124–SNAI2 signaling regulates epicardial EMT and myocardial development, with PKR1 loss producing CHD-like defects [59]. METTL3-dependent m6A methylation is required for normal cardiac morphogenesis [60], and VCL Y822 phosphorylation supports postnatal adhesion remodeling needed for later cardiac function [61]. Together, these findings link postnatal maturation to the progressive loss of cardiac regenerative capacity.

## Two-Dimensional PSC Differentiation Models

Two-dimensional (2D) models are useful for gaining insight into CHD phenotypes and mechanisms, and for complementing developmental studies in model organisms such as mice, zebrafish, and flies. Pluripotent stem cells, including mouse and human embryonic stem cells (mESCs and hESCs), and human induced pluripotent stem cells (hiPSCs), can be differentiated into a 2D monolayer of mesendoderm, cardiac mesoderm, cardiac progenitors, and cardiomyocytes, making them useful for testing CHD gene effect on lineage commitment, signaling, and phenotypes during cardiomyocyte differentiation.

Recent studies using 2D cardiac differentiation models have provided new insights into early lineage commitment. During human cardiac differentiation, GATA6 haploinsufficiency disrupted mesendoderm and lateral mesoderm specification, defects that were partially rescued by modulating WNT/BMP signaling during the first 48 h of differentiation [62]. Gain- and loss-of-function study of GATA4 in Xenopus explants and hiPSC-derived cardiomyocytes identified TBX2 and PRDM1 as key downstream targets that promote cardiomyocyte specification and suppress endothelial-lineage specification, respectively [63]. Other studies have identified regulators beyond canonical cardiac transcription factors. For example, NONO loss disrupted early hiPSC cardiomyocyte differentiation by reducing HOXA1 occupancy and compromising WNT activation [64]. Knockdown of the gene encoding the RNA-binding protein SAMD4A reduced hESC proliferation, cardiac mesoderm formation, and cardiomyocyte function, while FGF2 supplementation rescued proliferation defects, connecting RNA-binding protein activity to FGF2/AKT-dependent cardiac lineage specification [65]. Together, these studies demonstrate how 2D models can be used to dissect cardiac differentiation and lineage commitment.

2D differentiation models are also useful for CHD modeling and identifying therapeutic interventions. A CHD-associated GATA4 p.Arg284His variant in hiPSC-derived cardiomyocytes successfully captured the gene expression and electrophysiological defects and identified potential therapeutic targets [40]. The Seidman lab identified rare SMAD2 variants in a CHD cohort and used hiPSCs to model these mutations. SMAD2 haploinsufficiency altered chromatin accessibility, dysregulated SMAD2-regulated genes, reduced interaction with transcription factors at promoters of cardiovascular development genes, and contributed to the reclassification of SMAD2 variants of uncertain significance [66]. Further, using directed atrial and ventricular hiPSC differentiation in a *TBX5* dosage model, combined with cellular, electrophysiologic, chromatin-accessibility, and gene-regulatory readouts, we showed that reduced TBX5 dosage altered atrial cardiomyocyte identity and function [67]. Together, these studies support the value of 2D differentiation models in understanding CHD etiologies.

The strengths of 2D differentiation models are temporal control, human allele-aware perturbation, scalability, and compatibility with molecular and functional readouts. 2D systems facilitate modeling heterozygous variants, missense alleles, dosage-related mechanisms, and haploinsufficiency, as shown by recent SMAD2, TBX5, and GATA4 variant studies [40, 66, 67]. They also allow direct rescue experiments, including WNT/BMP modulation in GATA6-mutant hESCs, FGF2 rescue in SAMD4A-deficient cells, antioxidant reversal of serine-pathway effects, and NLR pathway inhibition in MAPK1-mutant cells [62, 65, 68, 69]. Finally, 2D models are scalable for CRISPR or siRNA screens and can be paired with metabolic and functional characterization, making them efficient platforms for connecting CHD variants to developmental mechanisms [70].

The limitations of 2D cultures are that they simplify the spatial and mechanical context of the developing heart, so they cannot fully capture morphogenesis, tissue-tissue interactions, hemodynamic cues, or the multicellular architecture that shapes CHD phenotypes in vivo. They are also sensitive to technical and biological variability, including genetic background, clone-to-clone variation, passage number, genome-editing effects, and differentiation conditions. While 2D models are ideal for rapid, allele-aware tests of early gene function and cell-autonomous phenotypes, other models, including 3D models, cardiac organoids, and in vivo mouse models, are needed to validate observations from 2D models.

## Three-Dimensional Model Systems

Three-dimensional (3D) differentiation models offer a way to study early human heart development in a more spatially organized and multicellular context. By allowing cardiac cells to self-organize, interact with other cell types, and respond to extracellular matrix cues, these systems can uncover developmental phenotypes that may be missed in 2D culture.

Gastruloids model developmental stages preceding heart formation and are valuable for studying CHD genes that act during cardiac mesoderm specification. Human and mouse gastruloids generate spatially organized mesodermal populations and have revealed that early cardiac phenotypes often arise through non-cell-autonomous mechanisms involving WNT signaling and cross-lineage interactions [71–73]. However, their developmental outcomes are highly sensitive to stem cell heterogeneity and culture conditions, emphasizing the need for optimized differentiation protocols [74–76]. In contrast, cardioids and chamber-like organoids recapitulate key aspects of cardiac morphogenesis, including cavitation, tissue architecture, and multicellular interactions. Recent reports show that geometrical confinement, matrix stiffness, cell adhesion, and extracellular matrix composition collectively regulate chamber formation, endothelial organization, and contractile maturation, providing increasingly physiological models of early heart development [77–79]. Furthermore, advances in 3D bioprinting help generate more mature, perfusable, chambered heart tissues that more closely resemble early heart development and provide sophisticated platforms for understanding CHD mechanisms [80–82].

Cardioids are powerful models for studying heart development. Recent studies show that geometrical confinement and coordinated WNT, BMP, retinoic acid, and FGF signaling regulate mesoderm-endoderm patterning, cavitation, contractile maturation, and endothelial-cardiomyocyte interactions, highlighting that tissue geometry and signaling cues are fundamental determinants of cardiac development, rather than just culture conditions [83, 84]. Mixed tissue 3D models further enhance developmental complexity by incorporating multiple cardiac cell types. Epicardium-myocardium organoids with sympathetic neuron spheroids recapitulate neurovascular organization, while heart macrophage assembloids reveal that tissue-resident macrophages evolve alongside the developing myocardium to regulate extracellular-matrix remodeling, electrical conduction, and immune signaling [85–87]. Complementary epicardial organoids and sinoatrial node cardiac assembloids model cardiomyocyte-epicardium interactions and pacemaker-to-atrial conduction, expanding the utility of organoid systems to investigate CHD and conduction system abnormalities [88–90].

Three-dimensional models of cardiac differentiation provide significant advantages over 2D culture by recapitulating tissue architecture, multicellular interactions, and biochemical cues that are essential for heart development. Organoids and engineered cardiac tissues support studies of cardiac chamber morphogenesis, endothelial-myocardial signaling, immune-cardiac interactions, and electrophysiological parameters, while patient-specific models have uncovered disease-specific mechanisms that are typically not captured in 2D systems. These advantages make 3D models useful for validating CHD-specific genes identified from human genetics or 2D differentiation studies.

Despite these advantages, current 3D models remain limited by developmental immaturity, variability, and lack of standardization. Different organoid platforms represent distinct developmental stages and differ in lineage composition, maturation, and geometry, making cross-platform comparisons challenging and requiring benchmarking against embryonic or fetal developmental reference datasets. Furthermore, differentiation outcomes are highly sensitive to culture conditions, and strategies to improve maturation often face reproducibility and scalability issues. Therefore, 3D cardiac models are most useful when combined with quantitative functional assays and validation in embryonic or in vivo systems [91–94].

## Genome Editing and Perturbation Methods

Genome editing converts CHD genomic discoveries into testable mechanisms. Sequencing identifies coding and noncoding variants, but edited models determine whether these variants perturb cardiogenesis. Introducing, correcting, deleting, or transcriptionally modulating loci in isogenic hiPSCs, cardiomyocyte differentiation systems, zebrafish, Xenopus, and mouse embryos links alleles to altered lineage specification, calcium handling, enhancer activity, morphogenesis, and gene-regulatory programs [95–98].

Prime editing (PE) and base editing (BE) model single-nucleotide variants underlying many pathogenic CHD alleles [97, 99, 100]. PE uses pegRNA-directed “search-and-replace” editing to install substitutions, insertions, or deletions without double-strand breaks, enabling heterozygous ESC/iPSC models and allele-specific correction for variants of uncertain significance [98, 99]. Correction of a heterozygous Nkx2-5 R52G mutation in mouse embryos restored atrial septation and downstream gene expression, demonstrating precise allele repair [96]. Base editors install transition mutations without donor templates, but bystander editing can confound interpretation [97, 100]. Isogenic pairs remain the gold standard for assigning cellular phenotypes, including altered calcium handling in GATA4 variants, to specific mutations [40, 98].

CRISPR–Cas9 enables scalable perturbation through knockout, CRISPRa/i, epigenome editing, transcriptome editing, and genome-wide sgRNA libraries [101–108]. New platforms include CRISPR-StAR, which uses internal controls within sgRNA-containing cells [109], saturation genome editing for variant-effect mapping, including MYBPC3 disease alleles [110], and patient-sequencing-informed in vivo Xenopus screens [111]. AAV-mediated delivery enables tissue-specific in vivo screening through engineered tropisms [112, 113], while Perturb-seq links CRISPR perturbation to single-cell transcriptomes for regulatory-network reconstruction [114]. Future screens will integrate multi-omics and machine learning to predict genotype–phenotype relationships [115].

Noncoding variants are a major frontier because many CHD cases lack a coding diagnosis [116–118]. Gata4 and Hand2 studies reveal enhancer redundancy and conditional disease-modifier effects [119], while lentiMPRA screens identify ncDNVs that disrupt activator motifs or create ectopic enhancer activity in iPSC-cardiomyocytes [118]. lncRNAs including HBL1, Upperhand, and Handsdown regulate differentiation timing and Hand2-locus activity [117].

Reporter knock-ins enable lineage tracing and scoreable phenotypes [98, 120], and epitope tagging supports chromatin-occupancy assays such as CUT&RUN [39]. Yet mosaicism, off-target effects, clonal epigenetic bias, editing efficiency, dosage sensitivity, and timing remain interpretive challenges [97, 98, 121, 122]. Temporal-control systems such as AID/AID2 allow rapid, reversible protein depletion and have revealed stage-specific requirements for chromatin remodelers in differentiation and mouse development [123–128]. High-content readouts, including single-cell multiome perturbation profiling and scDynabar lineage recording, capture molecular, temporal, and phenotypic states at single-cell resolution [129, 130].

CRISPR knockout approaches are powerful but lack temporal control. Auxin-inducible degron systems address this limitation by enabling rapid, reversible degradation of tagged proteins after auxin treatment while preserving transcription and translation [123–127]. AID2 improved the original system by reducing basal degradation, lowering ligand requirements, and accelerating degradation through a mutant TIR1 receptor and 5-Ph-IAA [124]. Degron studies have revealed stage-specific gene requirements, including BRM during mesoderm differentiation but not later stages [126], and their extension to mouse embryos and adult tissues supports temporally precise in vivo studies [128].

Functional genomics is moving beyond survival, proliferation, or reporter activity toward high-content molecular and cellular phenotyping. Single-cell multiome perturbation combines CRISPR perturbation with transcriptomic and chromatin-accessibility measurements, allowing for enhanced interpretation of gene-regulatory effects [129]. scDynabar integrates dynamic CRISPR barcoding with single-cell transcriptomics, linking gene expression to evolving states over time [130]. Together, these approaches make CRISPR screening a multidimensional platform for characterizing gene-regulatory networks at single-cell resolution.

Careful experimental design is essential for interpreting genome-editing and CRISPR-screening studies. In vivo perturbations can produce mosaic editing and variable efficiencies, complicating phenotypic analysis and reducing statistical power [114, 129]. Off-target effects remain a concern despite improved guide design, making validation with independent sgRNAs and complementary approaches important [121, 122]. Gene dosage, perturbation timing, and model context can strongly influence outcomes. Rescue experiments establish causality, while validation across cell lines, organoids, animal models, and human-derived systems distinguishes conserved mechanisms from model-specific artifacts [111, 114, 129].

## Conclusions and Future Directions

CHD research has entered an accelerated phase in which descriptive catalogs of genes, variants, cell states, and developmental trajectories are increasingly being used to design mechanistically informative functional experiments. Single-cell, spatial, genomic, and imaging studies have produced detailed maps of cardiac development, but the next major challenge is to determine which candidate genes and regulatory elements are causal, when and in which cell types they act, and how their disruption produces specific structural or functional phenotypes. Meeting this challenge will require integrated, multi-model strategies that combine the genetic precision of human pluripotent stem cell systems with the physiological relevance of engineered three-dimensional in vitro systems and in vivo mammalian models.

This transition is being dramatically accelerated by genome-editing technologies. CRISPR knockout, CRISPRi/a, base editing, prime editing, inducible degron systems, and lineage-recording approaches now allow investigators to perturb genes, alleles, regulatory elements, and proteins with unparalleled precision and temporal control. At the same time, high-throughput pooled assays are being actively refined and disseminated. As these approaches become more robust in stem cells, organoids, and in vivo systems, they will continue to accelerate the functional annotation of CHD-associated genes and noncoding variants, moving the field from one-gene-at-a-time validation toward systematic genotype–phenotype mapping.

Recent developments in artificial intelligence will undoubtedly further reshape this landscape. In the near term, AI-powered tools will accelerate data generation by greatly simplifying experimental design and execution, including activities such as vector design, guide selection, perturbation-library construction, image analysis, quality-control workflows, and bioinformatics. Over the longer term, AI models capable of synthesizing large, high-dimensional datasets across assays, species, and experimental systems may help identify developmental relationships, prioritize mechanisms, and generate new hypotheses. High-impact future studies will likely pair AI-assisted large-scale data analysis with rigorous genome-editing-based functional experiments across complementary model systems.

## Key References

- Ranade SS, Li F, Whalen S, Pelonero A, Ye L, Huang Y, Brand A, Nishino T, Mital R, Boileau RM, Koback F, et al. Myocardial reprogramming by HMGN1 underlies heart defects in trisomy 21. Nature. 2025 Nov 27;647(8091):979 − 87.
  - ⚬ This manuscript is a clear embodiment of the review’s central message: it combines human pluripotent stem cells, CRISPR screening, single-cell analysis, and mouse rescue experiments to identify a dosage-sensitive gene function and explain a specific CHD mechanism.
- Xiao F, Zhang X, Morton SU, Kim SW, Fan Y, Gorham JM, Zhang H, Berkson PJ, Mazumdar N, Cao Y, Chen J, et al. Functional dissection of human cardiac enhancers and noncoding de novo variants in congenital heart disease. Nature genetics. 2024 Mar;56(3):420 − 30.
  - ⚬ By functionally testing thousands of noncoding variants from CHD trios, this study directly addresses the “noncoding frontier” highlighted in our review, and demonstrates how high-throughput functional genomics assays can leverage descriptive genetic data to identify experimentally supported variant effects.
- Jin L, Park C, Neelakantan S, Karnik S, Dey A, Fineman S, Nandwani R, Gil CJ, Hwang B, Cadena MA, Sridhar V, et al. Modeling early human heart development using an iPSC-based 3D bioprinted model of embryonic heart tube. Nature Communications. 2026 Apr 24.
  - ⚬ The perfusable, multilayered embryonic heart-tube model introduces tissue geometry, extracellular matrix organization, and hemodynamic flow that conventional 2D cultures lack. It represents a strong example of 3D platforms becoming sufficiently sophisticated for mechanistic studies of human cardiac development.

## Data Availability

No datasets were generated or analysed during the current study.

## Declarations

### Use of Artificial Intelligence

Artificial intelligence (AI) tools were used for language and grammar editing. All content was reviewed and approved by the authors, who take full responsibility for the final manuscript.
